# Design of a Wearable Fingertip Haptic Device for Remote Palpation: Characterisation and Interface with a Virtual Environment

**DOI:** 10.3389/frobt.2018.00062

**Published:** 2018-06-12

**Authors:** Antonia Tzemanaki, Gorkem Anil Al, Chris Melhuish, Sanja Dogramadzi

**Affiliations:** Bristol Robotics Laboratory, University of the West of England, Bristol, United Kingdom

**Keywords:** surgical robotics, tele-operation, haptics, virtual environments, mechatronics

## Abstract

This paper presents the development of a wearable Fingertip Haptic Device (FHD) that can provide cutaneous feedback via a Variable Compliance Platform (VCP). The FHD includes an inertial measurement unit, which tracks the motion of the user’s finger while its haptic functionality relies on two parameters: pressure in the VCP and its linear displacement towards the fingertip. The combination of these two features results in various conditions of the FHD, which emulate the remote object or surface stiffness properties. Such a device can be used in tele-operation, including virtual reality applications, where rendering the level of stiffness of different physical or virtual materials could provide a more realistic haptic perception to the user. The FHD stiffness representation is characterised in terms of resulting pressure and force applied to the fingertip created through the relationship of the two functional parameters – pressure and displacement of the VCP. The FHD was tested in a series of user studies to assess its potential to create a user perception of the object’s variable stiffness. The viability of the FHD as a haptic device has been further confirmed by interfacing the users with a virtual environment. The developed virtual environment task required the users to follow a virtual path, identify objects of different hardness on the path and navigate away from “no-go” zones. The task was performed with and without the use of the variable compliance on the FHD. The results showed improved performance with the presence of the variable compliance provided by the FHD in all assessed categories and particularly in the ability to identify correctly between objects of different hardness.

## 1. Introduction

Master-slave robotic systems have found use in many applications ranging from games industry, assistive technologies and medicine (practicing or training) (e.g., in neuromuscular rehabilitation (Iqbal et al., 2014) or in surgery (Hagn et al., 2010; Tzemanaki et al., 2014; Intuitive Surgical, 2017) to areas where safety issues prevent use of autonomous robots such as in underwater environments, space exploration or nuclear industry (Nagatani et al., 2013; Kulakov et al., 2015; García et al., 2017).

Tracking or replicating hand/arm motion is a central part of tele-operation. We interact with our environment mainly using vision and touch. Our hands, with their complex structure, high dexterity and precise manipulation ability are critical instruments in recognising shape, stiffness and weight of an object (Achibet, 2015). Their high touch sensitivity is achieved due to mechanoreceptors embedded in the skin, which aid the detection of vibration, stretching and cutaneous stimuli.

The addition of haptics in tele-operation can add value and improve the performance of the user, for example in minimally invasive surgery (MIS) or microsurgery applications as supported by Abushagur et al. (2014). Contrary to techniques used in open surgery or even manual minimally invasive surgery (laparoscopy), one of the most frequent criticisms of robot-assisted MIS (R-A MIS) systems is their lack of haptic feedback (Abushagur et al., 2014). Use of master-slave systems requires extensive training to gain dexterity and efficiency (Ma et al., 2014). Practicing on actual R-A MIS systems is not easily available when junior surgeons start their training as surgical assistants. Virtual reality (VR) environment applications can be a cost-efficient substitute that accelerates the initial phases of training. Simulators including e.g., virtual pick and place tasks or even a simulated patient’s abdomen are already used in R-A MIS training or anatomy learning among others (van der Meijden and Schijven, 2009; Vander Poorten et al., 2012; Meng et al., 2013). The addition of haptic feedback in VR environments creates more realistic scenarios, while providing trainees with a safe environment in which they can develop their skills (Lemole et al., 2007; Kirkman et al., 2014).

Haptic feedback is commonly categorised as kinaesthetic and cutaneous/tactile feedback. Various haptic devices are commercially available e.g., the Geomagic Touch haptic device (USA, formerly Sensable Phantom Omni) with a simple and safe design which made its use popular (Srinivasan and Basdogan, 1997). Such devices can be classified as grounded kinaesthetic (mainly) feedback devices that are portable but placed on a surface while the user operates their end-effector in 3D space. A Phantom Omni is used in the work by Li et al. (2015), where examined palpation and tumor identification using force feedback and Pseudo-Haptic Feedback (PHF) in a virtual environment. In this work, PHF is based in visual cues of virtual tissue deforming while the speed of the screen’s cursor is slowed down to give the impression of stiffness. PHF is a good alternative when a force feedback device is not present or possible, while it can also compliment and improve the results of haptic feedback devices (Li et al., 2015).

Similar devices include the Falcon (Novint, USA) or Sigma.7 (Force Dimension, Switzerland), both with a parallel robot configuration as opposed to the serial configuration of Geomagic Touch. However, their prices can be high (Pacchierotti et al., 2017) and their workspace is usually restricted.

Moreover, grounded haptic devices have been designed specifically to support fingers. For example, MasterFinger-2 is a 6-DOF (Degree Of Freedom) haptic interface which can be operated using the index finger and the thumb to grasp virtual objects (Monroy et al., 2008, Pacchierotti et al., 2012). Such systems can provide both kinaesthetic and cutaneous feedback to the users, however, their workspace is limited and they are not meant to be portable.

Wearable Haptic Devices (WHDs) such as Hand Exoskeletons (HE) can provide more freedom of movement, mimic the hand movements of the operator and potentially remove the cognitive gap in tele-operation (van der Meijden and Schijven, 2009). An example WHD is the combination of Cyberglove and CyberGrasp (CyberGlove Systems, USA) which provides force feedback by pulling the fingertips via cables. The glove by Park et al. (2016) is also using cables: one to measure the position of each finger and one to exert force on the fingers. Other WHDs utilise rigid force transmission mechanisms attached to the hand to exert force on the fingers such as the one by Fontana et al. (2013) (thumb and index finger) or DEXMO exoskeleton by Gu et al. (2016). Despite DEXMO’s high motion accuracy, its major disadvantage is its ability to generate only binary haptic feedback (Gu et al., 2016).

HEs are made using soft or rigid materials, covering all or some of the fingers. Some exoskeleton designs are bulky while some cover just fingertips, which can be especially effective for tactile applications and controllable cutaneous feedback (Pacchierotti et al., 2017) and can be classified according to the cutaneous sensation that they provide: normal indentation, tangential motion, lateral skin stretch or vibration (Pacchierotti et al., 2017). An example of use of vibration for haptic feedback is the device by Maereg et al. (2017) with five vibro-tactile actuators, one for each fingertip of the user. Although wearable devices usually provide cutaneous stimuli, with most of the kinaesthetic feedback missing (Prattichizzo et al., 2013), it is possible to compensate for this deficiency without significant performance degradation (Pacchierotti et al., 2014).

Normal indentation is achieved by one or more moving parts that emulate contact with a soft/hard material or give a sense of curvature or pressure to the fingertip. Furthermore, lateral motion with respect to the fingertips can apply cutaneous feedback to fingertip. For example, the combination of the two methods (normal and lateral) has been utilised via three motors, cables and a parallel mechanism by Prattichizzo et al. (2013) and Pacchierotti et al. (2014) or by a serial mechanism wrapped around the finger actuated by a motor and a voice coil by Gabardi et al. (2016) for surface exploration. In the work by Pacchierotti et al. (2014), the motors adjust the length of cables using position encoders to move the platform towards the fingertip. A force sensor is attached to the platform’s centre to measure forces perpendicular to the fingertip.

Kim et al. (2016) propose a similar haptic fingertip device with the addition of four Inertial Measurement Units (IMU) sensors to track the palm and index finger in a virtual environment. Although these platform devices can be used in different scenarios, the end-effectors are constantly in contact with the fingertip, which does not allow the possibility of intermittent touch with virtual objects. In this respect, Chinello et al. (2015) presented a wearable fingertip device with two platforms in parallel configuration. Three servo motors are fixed to the upper part of the device and a mobile platform exerts forces to the volar skin surface of the fingertip. Motors actuate three legs connecting these two parts to render forces from the virtual environment. A virtual environment is used for testing also in the work by Maereg et al. (2017) where tracking is done via a LeapMotion controller and PHF is also explored by visualising displacement in the virtual environment.

In addition to applying force to a fingertip using moving platforms, the devices that are designed for lateral skin stretch can be also used to exert normal forces on the fingertip. Minamizawa et al. (2007) developed a wearable fingertip device to exert tangential and normal cutaneous feedback. When the two motors of the device rotate in opposite directions, the belt exerts vertical stress. Equally, rotation in the same direction results in shearing stress. A similar fabric based WHD by Bianchi et al. (2016) emulates different levels of softness by stretching the fabric across the fingertip, applying tangential forces.

A 3RSR parallel mechanism located under the finger as a moving platform, described by Leonardis et al. (2015), provides both position and orientation information. A fingertip delta type parallel mechanism has been designed by Schorr and Okamura (2017) that exerts normal, lateral and longitudinal skin deformation, with a maximum normal force of 2 N.

Murray et al. (2003) found that proportional haptic feedback, as opposed to binary feedback such as in the exoskeleton by Gu et al. (2016), can facilitate user performance. As discussed earlier, such devices can facilitate medical diagnosis or training on diagnosis (e.g., tumour detection), while it can also improve safety during precision operations by using haptic feedback as a warning (e.g., when navigating through a narrow space with “no-go” zones).

In this paper, we present the design of a Fingertip Haptic Device (FHD) for motion tracking and cutaneous haptic feedback that can be used to interact with a virtual environment in such a way that the user can gather information about the compliance as well as the hardness of different objects. The variable compliance in the FHD differs from previous work where fingertip indentations were achieved using objects of constant stiffness and shape or fabric materials that provide lateral deformation of the skin. Thus, variable compliance is designed to increase the sense of touch, and to provide the sense of touching soft or hard materials using a single device.

The FHD is 3D printed and comprises an IMU for motion tracking and a soft fingertip platform of adjustable compliance which is linearly actuated. The design of the mechanism is presented in Section 2, while its characterisation and testing are demonstrated in Section 3.1. To validate the developed mechanism, a series of experiments were carried out in a virtual environment where users had to complete a task using the FHD’s motion tracking system and haptic feedback. The results of this user study are presented in Section 3.2 and are discussed in Section 4, where a comparison between the various scenarios is made and conclusions are drawn.

## 2. Material and Methods

This Section presents the materials used for the FHD design as well as the methods used for motion tracking and interaction with a virtual environment. The haptic functionality of the FHD relies on the two features of its Variable Compliance Platform (VCP): the VCP can be inflated with air and linearly displaced so that it pushes against the fingertip. The combination of these can provide both the sense of the material’s hardness and of the normal force exerted on the fingertip. The forces generated by the VCP push against the user’s fingertip, allowing the user to passively palpate the material hardness.

### 2.1. Components of the FHD

**Figure 1:**
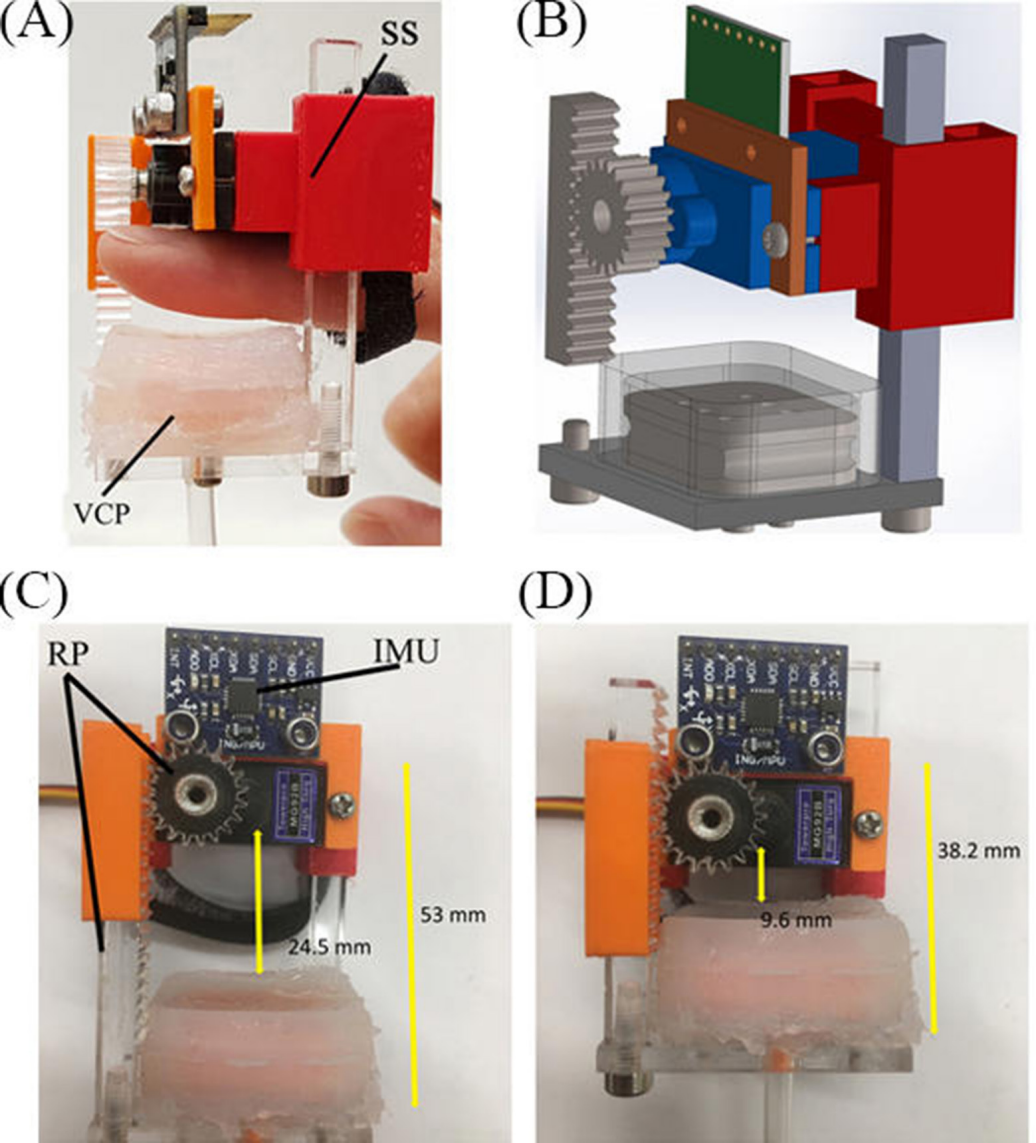
FHD: **(A)** side view with an index finger placed against the SS and held with a flexible strap; the RP mechanism lies behind the finger and the VCP below the finger, which can linearly move along the RP closer/farther from the finger, **(B)** Computer Aided Design drawing, **(C–D)** font views with the **(C)** minimum and **(D)** maximum VCP displacement.

Figure 1 shows the side and front view of the FHD, which consists of: (a) the VCP, (b) the Rack and Pinion (RP) mechanism and (c) the Support Structure (SS) with the IMU sensor. The RP mechanism adjusts the distance between the fingertip and the VCP. The FHD’s dimensions are 38.6 mm (width) x [38.2–53] mm (variable length due to the RP). The distance between the SS and the VCP range is 9.6–24.53 mm. The total weight of the FHD (including one motor, TowerPro MG92B) is 40 gr.

#### 2.1.1. VCP Design and Functionality

The 3D printed (Polylactic Acid filament) VCP has an area of 478.5 mm^2^ that corresponds to the average area of a fingertip as reported by Peters et al. (2002) (index finger, female average 360 mm^2^, male average 420 mm^2^).

**Figure 2:**
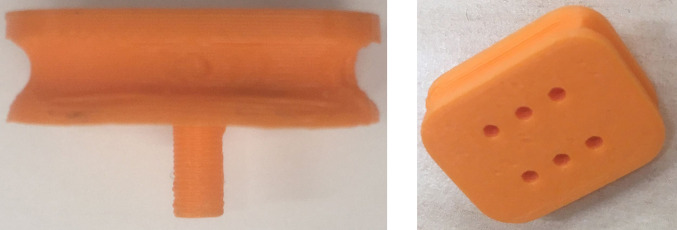
Rigid base of the VCP **(A)** side view, **(B)** isometric view.

**Figure 3:**
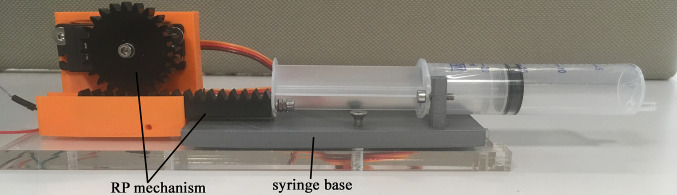
Syringe pump actuation system.

As discussed in the previous section, fingertip haptic feedback is often achieved by pressing rigid (Tsetserukou et al., 2010; Leonardis et al., 2015; Schorr and Okamura, 2017) or soft [e.g., belt systems by Minamizawa et al. (2007); Pacchierotti et al. (2014), dielectric elastomer actuators by Koo et al. (2008), Frediani et al. (2014)] objects either normal or lateral to the fingertip surface. However, these devices do not offer actual indentation sense because their compliance cannot be changed. Our hypothesis is that variable compliance in a haptic device can provide indentation and varied hardness/softness sense to the user. Consequently, the VCP consists of a rigid base (Figure 2) with its top surface covered by a layer of silicon rubber (DragonSkin, shore hardness 10 A, 475 psi) of 1 mm thickness. The lower surface (Figure 2A) of the VCP is connected to a syringe pump via a 7 mm diameter air tube (Figure 3). The VCP functionality is created by pumping air through 6 holes (Figure 2B) into the gap between its rigid base and the soft silicon membrane of the VCP.

The design of the syringe pump actuation system, shown in Figure 3, utilises an RP mechanism (part 1, Figure 3) and a 20 ml syringe (attached to part 2, Figure 3). The pinion is attached to a motor (Turnigy 1258 TG, stall torque of 1.17 Nm) and the rack moves the syringe along the horizontal axis (0.8 mm displacement per one degree of rotation). The maximum volume of air used for inflation of the VCP was 4 ml, equivalent to pressure of 5.17 kPa, measured using a pressure sensor (HSCSAAN015PDAA5, Honeywell, USA, range of ±103 kPa, accuracy of 0.25 kPa).

The extent of the VCP’s deformation when inflated with 4 ml of air is 25 mm, while index fingertip extent is 10.4 mm in average for women and 12.7 mm for men (Peters et al., 2002). The extent of the VCP is greater than the measured human fingertip because the contact area will be smaller when the soft membrane is inflated.

#### 2.1.2. RP Mechanism for Linear Displacement of the VCP

**Figure 4:**
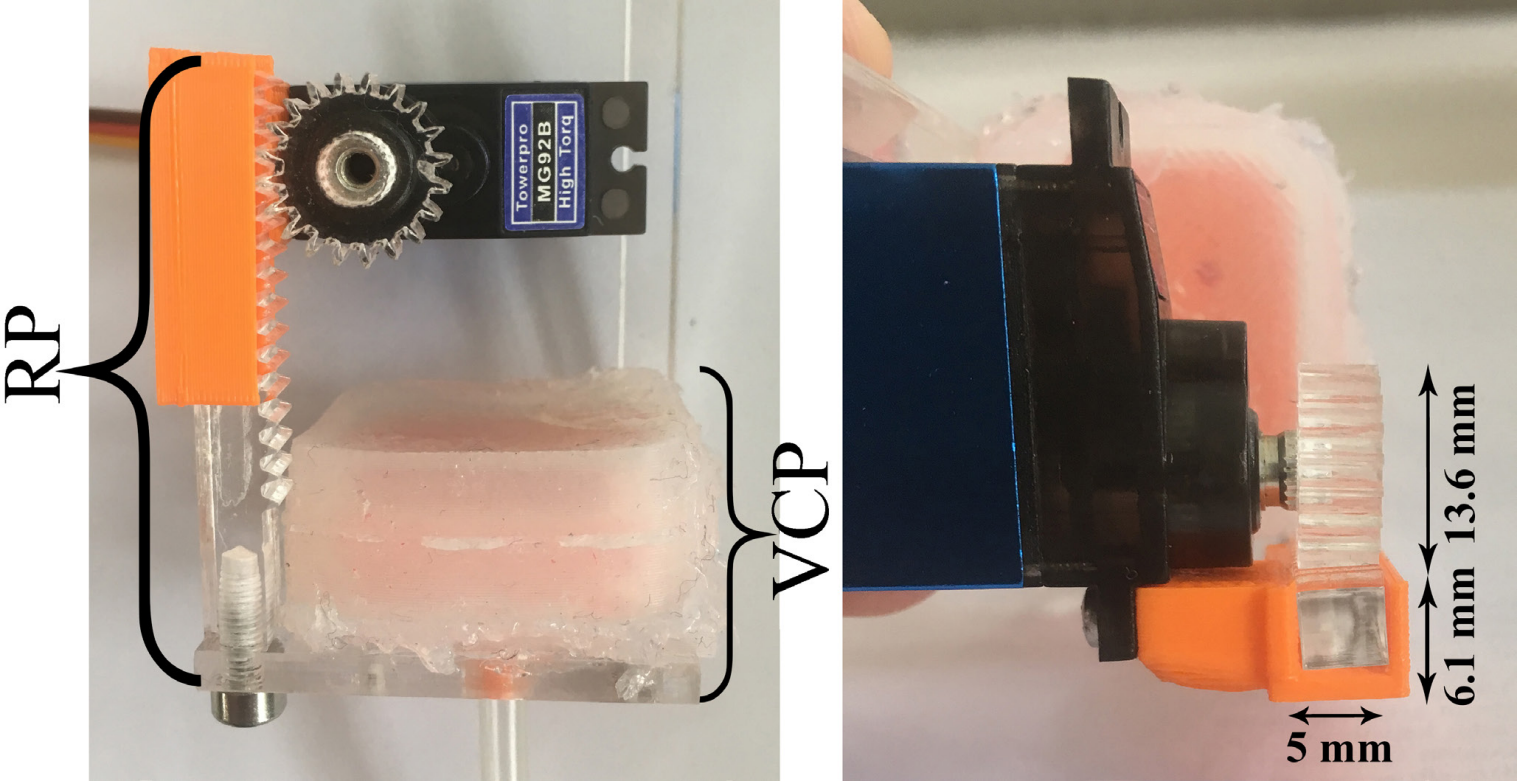
Linear mechanism of the VCP using an RP mechanism.

The chosen mechanism provides linear displacement of the VCP towards the fingertip and control of the indentation of the inflated membrane. The rack length is 30 mm (other dimensions are shown in Figure 4). This design was preferred to a parallel mechanism (Pacchierotti et al., 2014; Leonardis et al., 2015) to keep the size of the FHD to the minimum.

The shaft of the motor (TowerPro MG92B, stall torque of 0.3 Nm) is directly attached to the pinion. The linear displacement δ of the VCP can be calculated as follows:

(1) $$\delta =\left(2\pi \frac{d}{2}\right)\frac{\theta }{360}$$

where θ is the angle of motor rotation and d is the diameter of the pinion. Due to the required teeth precision, both rack and pinion were laser-cut in acrylic.

The variable compliance is created by varying the pressure inside the VCP which is a function of the piston movement (x) and the movement of the RP (h). It can be approximated using (2):

(2) $$p=p_{0}+k_{p}\left(x+h\right)$$

where p is the pressure inside the VCP, p_0_ is the initial pressure, k_p_ is the air spring constant between the piston and the finger. The piston movement (x) is proportional to the air volume supply through the syringe. The perceived hardness will be tested by a range of combinations of x and h that will effectively create different indentations in the human finger. 

### 2.2. Motion Tracking and VR Environment

For the motion tracking of the user’s fingertip, an MPU 6050 (InvenSense, USA) IMU is used. The IMU is integrated with the 3D printed support structure of the FHD (Figure 1B). The IMU’s raw data are sent to an Arduino MEGA 2560 board via I^2^C.

The motion of the user is tracked and fed into a virtual environment created in Unity 3D. The user moves virtual objects in the 3D VR environment, which is realised using the IMU data, while interaction with virtual objects is emulated by the FHD by (a) moving the VCP using the RP and (b) inflating the VCP using the syringe pump actuation system.

### 2.3. User Studies

#### 2.3.1. Air Volume – VCP Linear Displacement Relation

**Figure 5:**
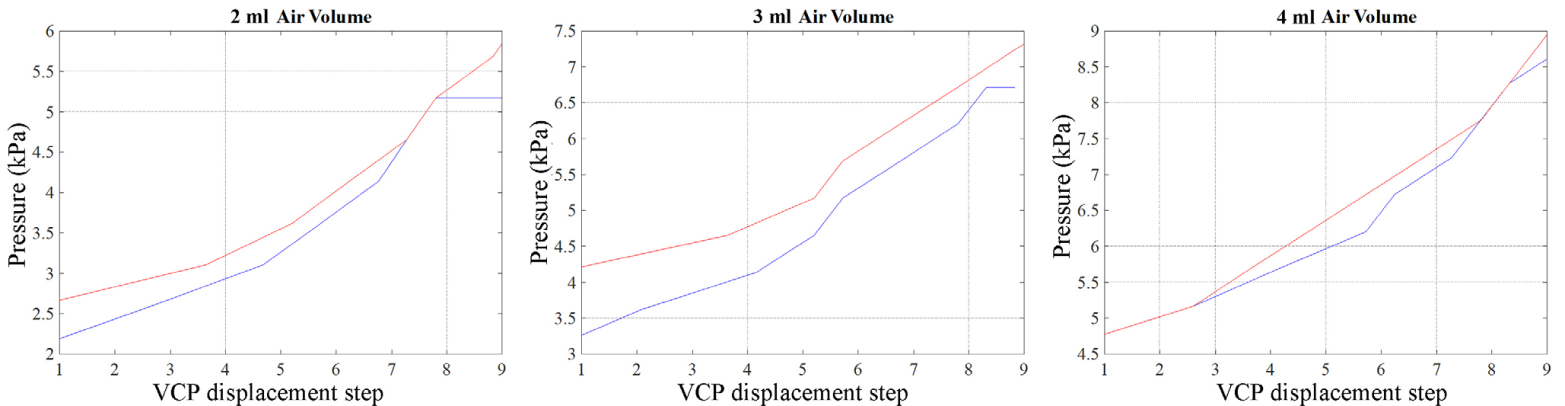
Pressure in the VCP vs maximum deformation of its surface.

Initial tests with 1 female and 1 male participant were carried out to establish a relationship between the air pressure and the linear displacement of the VCP. The users placed their index finger as shown in Figure 1A. The index finger was used as previous studies have shown that is intuitively used by humans for palpation (Konstantinova et al., 2017). After the VCP was moved to the point of initial contact with the fingertip, the motor rotated with a 5-degree step (0.52 mm of linear displacement of the VCP). The tests were carried out with 2 ml, 3 ml and 4 ml of air in the VCP, shown in the graphs of Figure 5 for the female (red line) and male (blue line) user. Each participant repeated the process 3 times, with the results being exactly repeatable, possibly including small errors due to the sensor’s resolution (0.25 kPa). Due to the different fingertip sizes, the maximum pressure in the VCP was overall higher for the female user. Maximum VCP displacement was 8.32 mm for the male user corresponding to a normal force of 3.85 N and 9.36 mm for the female with a normal force of 4.63 N. The normal forces exerted were measured when the fingertip was not present using a micro load cell (CZL635, Phidgets, 0–49 N range).

These tests provided initial results for further investigations of the resulting VCP pressure and combinations of the supplied air volume and its linear displacement along the rack axis. This study was limited to two participants and the dataset with the lower measured force for the male participant was adopted for all subsequent tests to pre-empt potentially uncomfortable high forces between the VCP and the fingertip.

#### 2.3.2. Perception of Hardness Using the FHD

In order to represent and assess the hardness levels of the FHD, a wider user study was carried out with 15 participants (18–34 years old, ratio of women/men 7/8, ratio of right/left dominant hand 13/2). The participants were asked to put the FHD on their dominant hand’s index fingertip and score the hardness of the touch on a scale of 1–5 (hard to soft). The experiments tested 10 different conditions created by varying the air volume inside the VCP as well as its linear displacement (and proximity to the fingertip). In one of the conditions, the VCP was not inflated while its linear displacement was 5.72 mm. The remaining 9 combinations are presented in Table 1. In this Table, “x” means that for that specific volume of air, the level of pressure could not be achieved. Each condition was tested 5 times by each participant in a randomised order after a short “training” session in which the participants could experience the different hardness levels of the FHD.

**Table 1:** Resulting pressure caused by different combinations of air volumes in the VCP and its linear displacement used to test different hardness levels for the FHD.

		Air volume in the VCP		
		2 ml	3 ml	4 ml
Resulting Pressure	3.5 kPa	1.1 (5.2 mm)	1.2 (2.08 mm)	x
	4.5 kPa	2.1 (7.28 mm)	2.2 (5.2 mm)	x
	5 kPa	3.1 (7.8 mm)	3.2 (5.72 mm)	3.3 (2.08 mm)
	7 kPa	x	4.2 (8.32 mm)	4.3 (6.76 mm)

These experiments have compared the hardness perception of different users for the same level of pressure at different volumes of air in the VCP. For example, 3.5 kPa can be derived at 2 ml of air and 5.2 mm displacement as well as at 3 ml of air and 2.08 mm displacement of the VCP. The experimental measurements that were used are the ones presented for the male participant of the previous experiment presented in Figure 5. Code names for each combination of air pressure and volume in the VCP that was used in this study, as well as the measured normal force exerted (micro load cell CZL635, Phidgets), are shown in Table 2.

**Table 2:** Corresponding exerted normal force for each condition of Table 1.

Condition	Normal Force (N)	Name in Table 1	Air Volume in the VCP (ml)	Resulting Pressure (kPa)
1	6.23	3.1	2	5
2	2.87	2.2	3	4.5
3	4.33	4.3	4	7
4	5.55	2.1	2	4.5
5	3.14	1.1	2	3.5
6	6.76	No air		
7	7.31	4.2	3	7
8	1.68	3.3	4	5
9	0.93	1.2	3	3.5
10	3.14	3.2	3	5

## 3. Results

This section presents the test results using the FHD that characterise its performance. Moreover, we have performed a series of experiments to assess user perception of the VCP hardness levels. All experiments were carried out in accordance with the recommendations of the University’s policy on research ethics, UWE Research Ethics Committee. The protocol was approved by the Faculty of Environment & Technology Research Ethics Committee. All subjects gave written informed consent in accordance with the Declaration of Helsinki.

### 3.1. Characterization of the FHD Components

#### 3.1.1. VCP Pressure and Deformation

**Figure 6:**
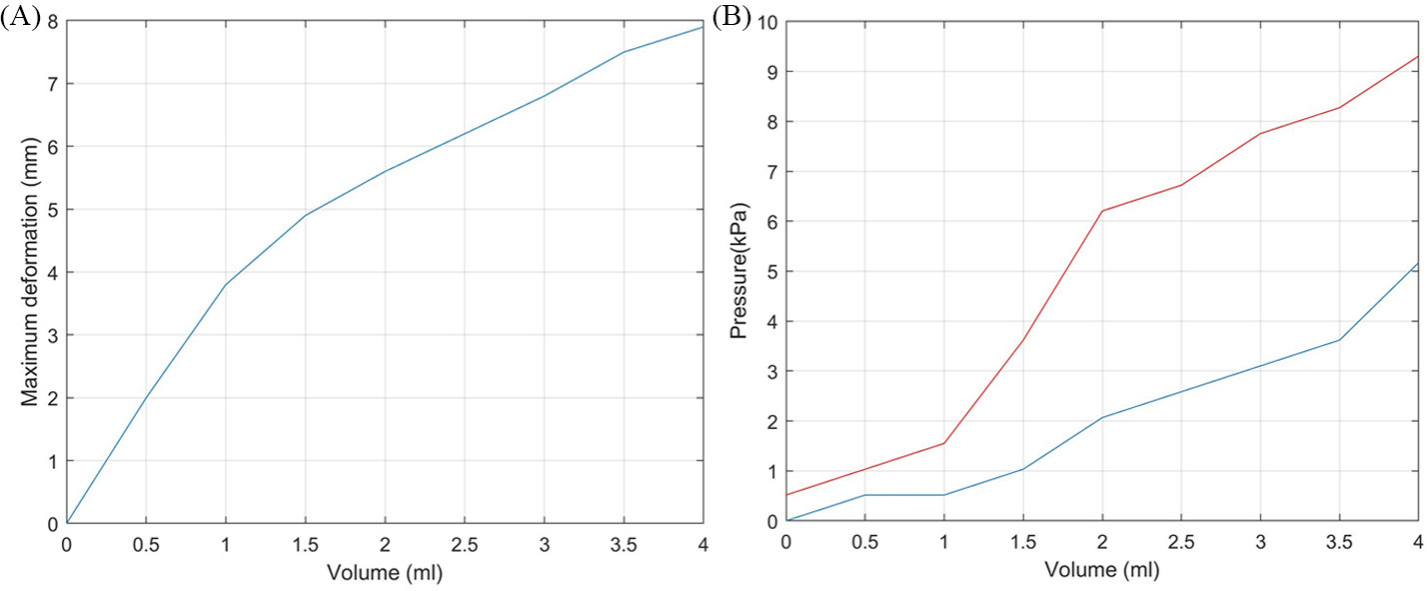
**(A)** Air volume (mm^3^) vs maximum VCP deformation and **(B)** Pressure in the VCP vs supplied air volume.

The maximum deformation of the VCP was measured 5 times using a high accuracy CCD laser displacement sensor (LK-G402, Keyence) while it was inflated with the volume of air in the range of 0–4 ml (Figure 6A) and without contact with the user’s finger. Above 1 ml, the VCP deformation increases slower with the volume.

**Figure 7:**
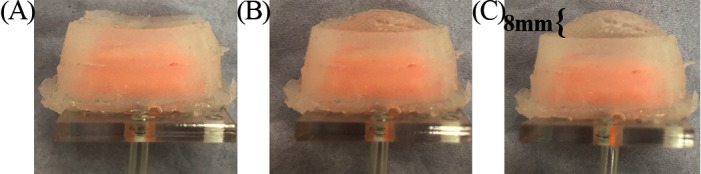
Inflation of the VCP with **(A)** no air **(B)** 2 ml and **(C)** 4 ml.

The pressure was recorded when the VCP was inflated (blue line, Figure 6B) and when it was inflated but with the index finger placed against the surface until contact with the rigid base of the VCP was felt by the user (red line, Figure 6B). A steep pressure change (1.5 to 6.2 kPa) occurs when the air volume increases from 1 to 2ml and when the fingertip applies pressure on the VCP. During this volume increase, the maximum deformation changes from 48 to 71% of its highest value, i.e., a 28% change, compared to a change of 48% for 0–1 ml. This means that when the VCP deforms with a high rate, the pressure increases slowly. Maximum pressure with the finger present is 9.3 kPa, compared to 5.1 kPa without the presence of a fingertip. Figure 7 depicts the actual VCP in non-inflated and inflated states. For example, the maximum deformation at 4 ml is 8 mm.

**Figure 8:**
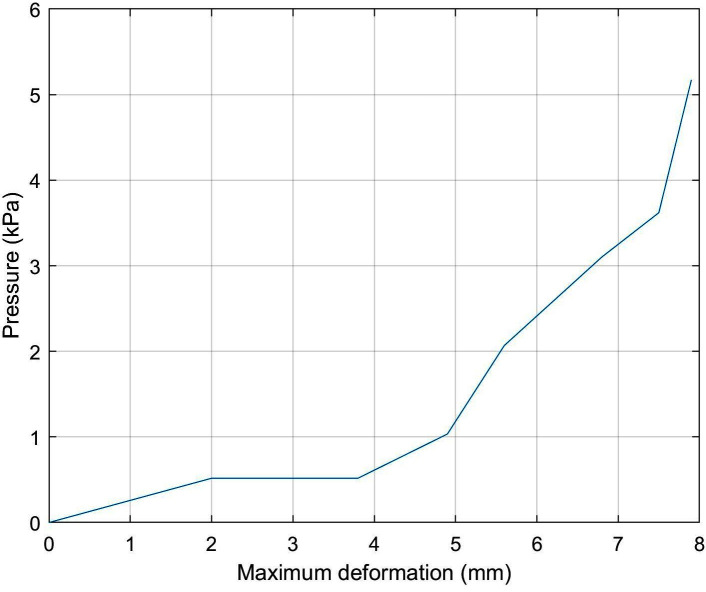
Pressure inside the VCP vs its maximum deformation.

Combining the two graphs in Figure 6, it is possible to identify a relationship between the pressure inside the VCP and its maximum deformation (Figure 8). The air pressure inside the VCP does not change when subjected to deformations between 2 mm (0.5 ml) and 3.8 mm (1 ml). This was assumed to occur due to the gap between the rigid (base) and flexible (membrane) part of the VCP.

#### 3.1.2. User Study Assessment of FHD Hardness

**Figure 9:**
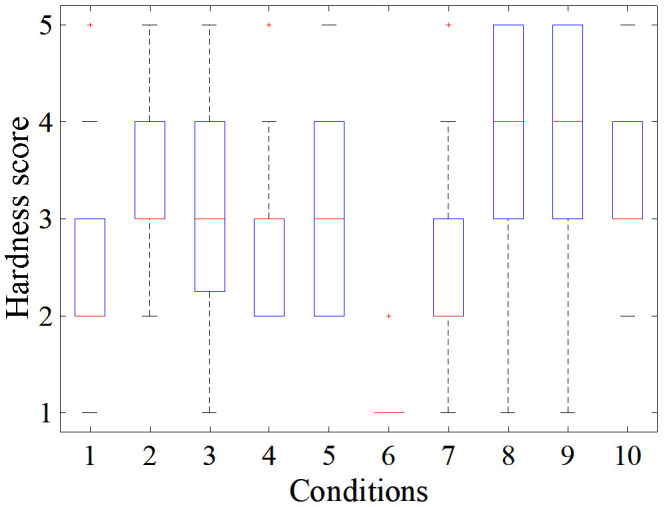
Distribution of participants’ response for each condition among a scale of 1–5 (hard to soft).

The box graph of Figure 9 illustrates how participants scored the hardness of the VCP for each condition. While the hardness of condition 6 (the VCP was not inflated) was evaluated with a score of “1” (hard), conditions 8 (4 ml of air) and 9 (3 ml of air) were evaluated as the softest (scores of “4” and “5”). In both conditions, the VCP moved by 2.08 mm and applied force to the fingertip of participants gently with the percentage of hardness score “4” and “5” being similar; however, the percentage of score “5” is higher in condition 9 (just above 35%), which suggests that for the same displacement, the VCP feels softer when filled with 3 ml of air. At 3 ml, the VCP is at medium capacity which makes it more compliant than at 2 ml or 4 ml. This is also seen when comparing conditions 2 (3 ml of air) and 5 (2 ml of air), for which the percentage of score “2” is under 20% and just above 35% respectively and hence condition 2 is considered softer.

The distribution of hardness score for conditions 1, 4 and 7 was between “2” and “3”. The percentage of score “2” of condition 1, 4 and 7 was approximately 50, 40 and 49% respectively, with condition 4 providing slightly softer feeling than conditions 1 and 7. This was expected as the VCP was displaced by 0.52 mm more (1 step) than in condition 4. A comparison between conditions 1 and 7 shows that in the latter, the VCP has 1 ml of air more and it is displaced by 1 step more than in condition 1. As the hardness score is similar for these conditions, this indicates that 1 step of increase in air volume cancels out 1 step of increase in displacement. Comparing conditions 5 (2 ml of air) and 3 (4 ml of air and 3 steps of displacement more than condition (5), their percentage of the combined score of “3” and “4” is similar. However, condition 5 had a more equal distribution between scores “2”, “3” and “4” than condition 3 which, as will be discussed later, prompted a more consistent response between participants.

**Figure 10:**
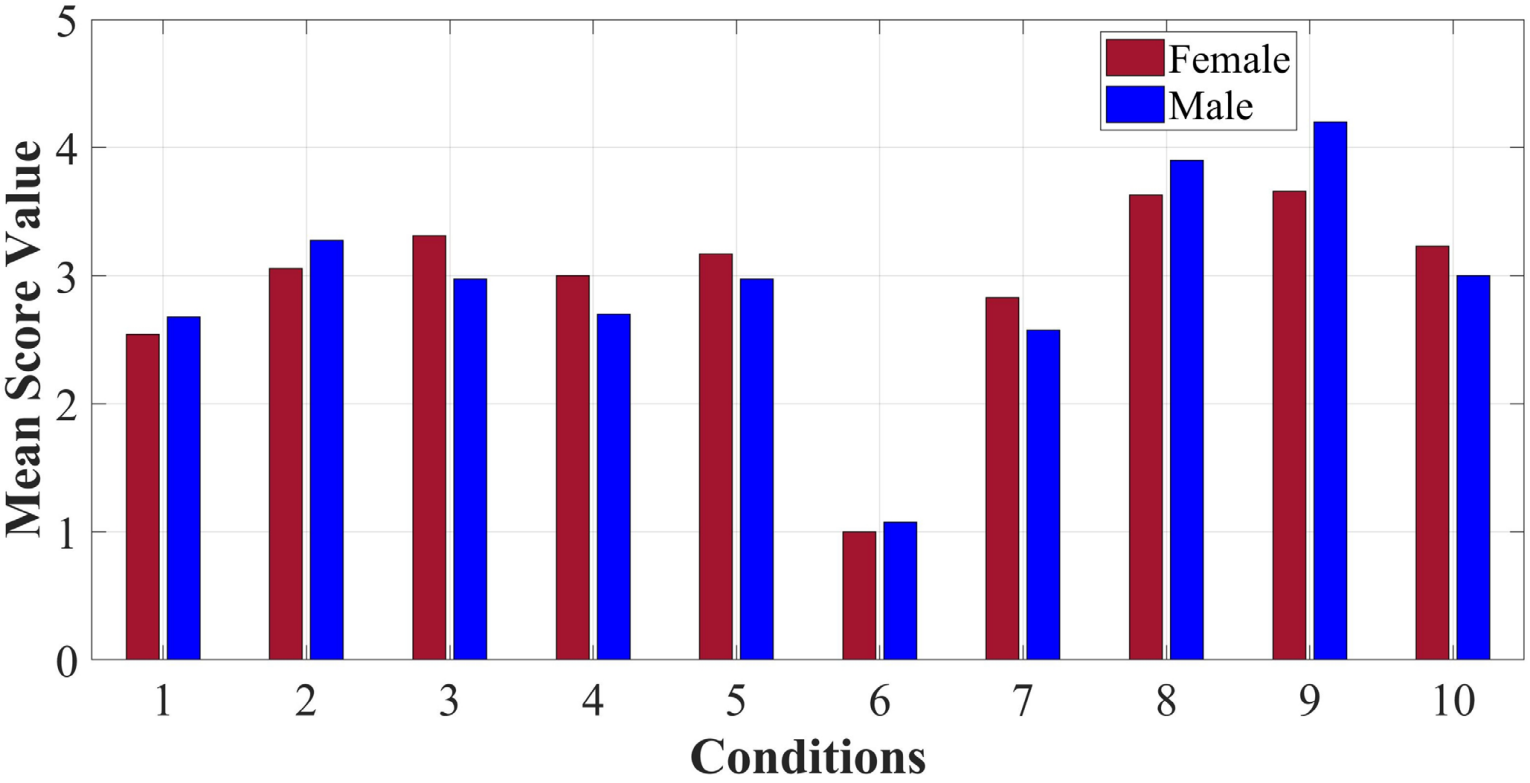
Mean scores for responses of women (red colour) and men (blue colour).

For conditions 2 and 10, the distribution was similar due to only 1 step of displacement difference between them, mainly between scores “3” and “4”, with “3” being the prevailing score. However, conditions 3 and 10 seem to have a clearer tendency towards a score of “3”, with condition 10 (3 ml of air) considered slightly softer. Finally, Figure 10 shows that there was no significant difference between responses of men and women, with SD for conditions 1–10 respectively: 0.07, 0.11, 0.17, 0.15, 0.1, 0.04, 0.13, 0.14, 0.27, 0.11 (mean of 0.128).

It is worth noting that user perception of hardness does not always correlate with the measured normal force exerted by the VCP. For example, condition 7 was considered softer than 6 despite the VCP exerting higher normal force in the former. This is due to the inflation of the VCP with 3 ml of air.

**Figure 11:**
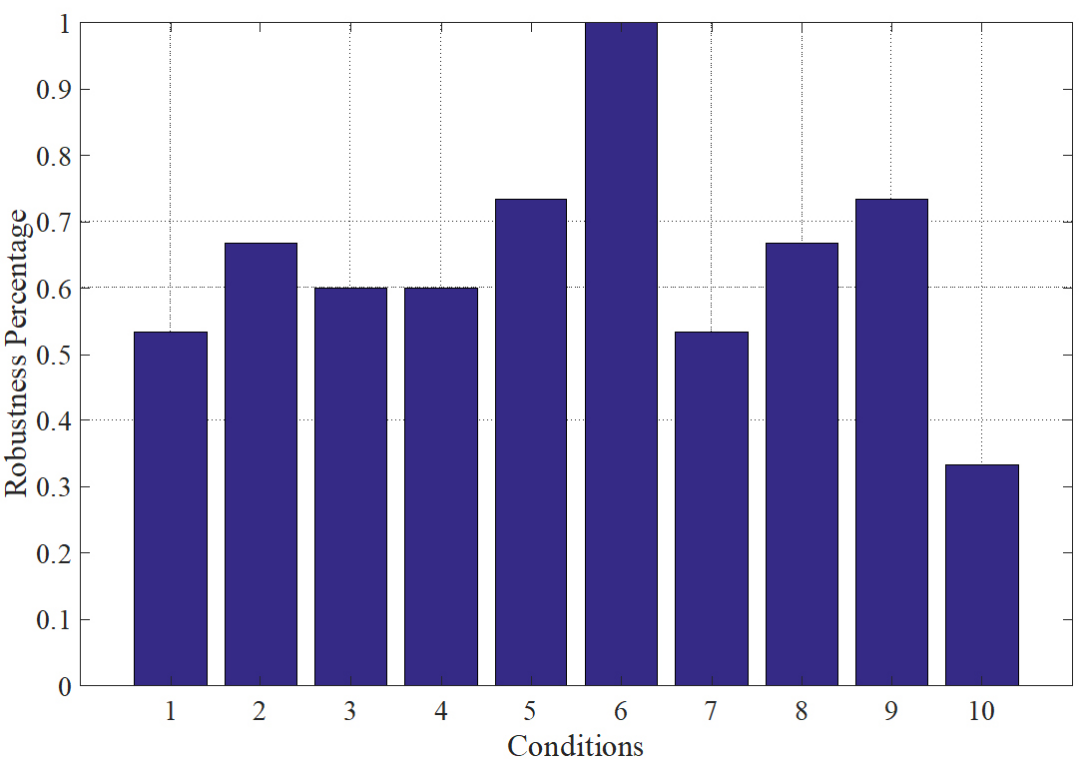
Percentage of consistency (robustness) between participants’ responses.

The 2nd column of Table 3 summarises the conditions that correspond to each score of the 1st column according to most of the participants’ answers. However, for conditions 1 and 10, the participants’ responses were not consistent (each condition was randomly repeated 5 times). For example, participant A scored condition 1 with “2, “3”, “4”, “2”, “3” across the 5 repetitions of the test, while participant B scored condition 5 with “2”,“3”, “3”, “2”, “2”. This would indicate that condition 5 receives more robust (consistent) responses than condition 1, as the participant appoints the same score to it more times (in this case, score “2” and “3”, instead of score “2”, “3” and “4”). Based on this criterion, conditions 2, 5, 6, 8 and 9 were the most robust, as shown in the 3^rd^ column of Table 3. Figure 11 shows the distribution of the “robustness percentage” of all conditions, determined by whether a participant’s set of (5) responses regarding a condition contained a maximum of 2 different scores (e.g., “2” and “3”).

**Table 3:** Results of user study: mapping each condition to a hardness score.

Score (1–5, hard-soft)	Condition with highest percentage	Conditions with consistent responses
“1”	6	6
“2”	1	5
“3”	10	2
“4”	8	8
“5”	9	9

 It must be noted that responses of different participants can vary for a given condition. For example, participant C scored condition 9 with “5”, “5”, “4”, “4”, “4” while participant D scored it with “3”, “3”, “2”, “2”, “2”. In the analysis of the data and the application in the experiments of the following section, this behaviour was still treated as robust. Despite such discrepancies and because the responses can only be subjective for each individual, it was considered important that conditions evoke consistent responses for each individual. Consequently, the 3rd column of Table 3 summarises the chosen robust conditions to be used in further studies of the FHD, while the 1st column shows their corresponding hardness level. The results show that the FHD can offer at least 5 different hardness levels and therefore it could realize various hardness levels of different objects in VR environment applications.

### 3.2. Implementation of the FHD - Path Following and Identification of Object Hardness

Based on the results of the previous user study and the experimental comparison between various combinations of the two features of the FHD (linear displacement and air volume of the VCP), the “robust” conditions of Table 3 were used to emulate different levels of hardness in a VR environment created in Unity 3D.

**Figure 12:**
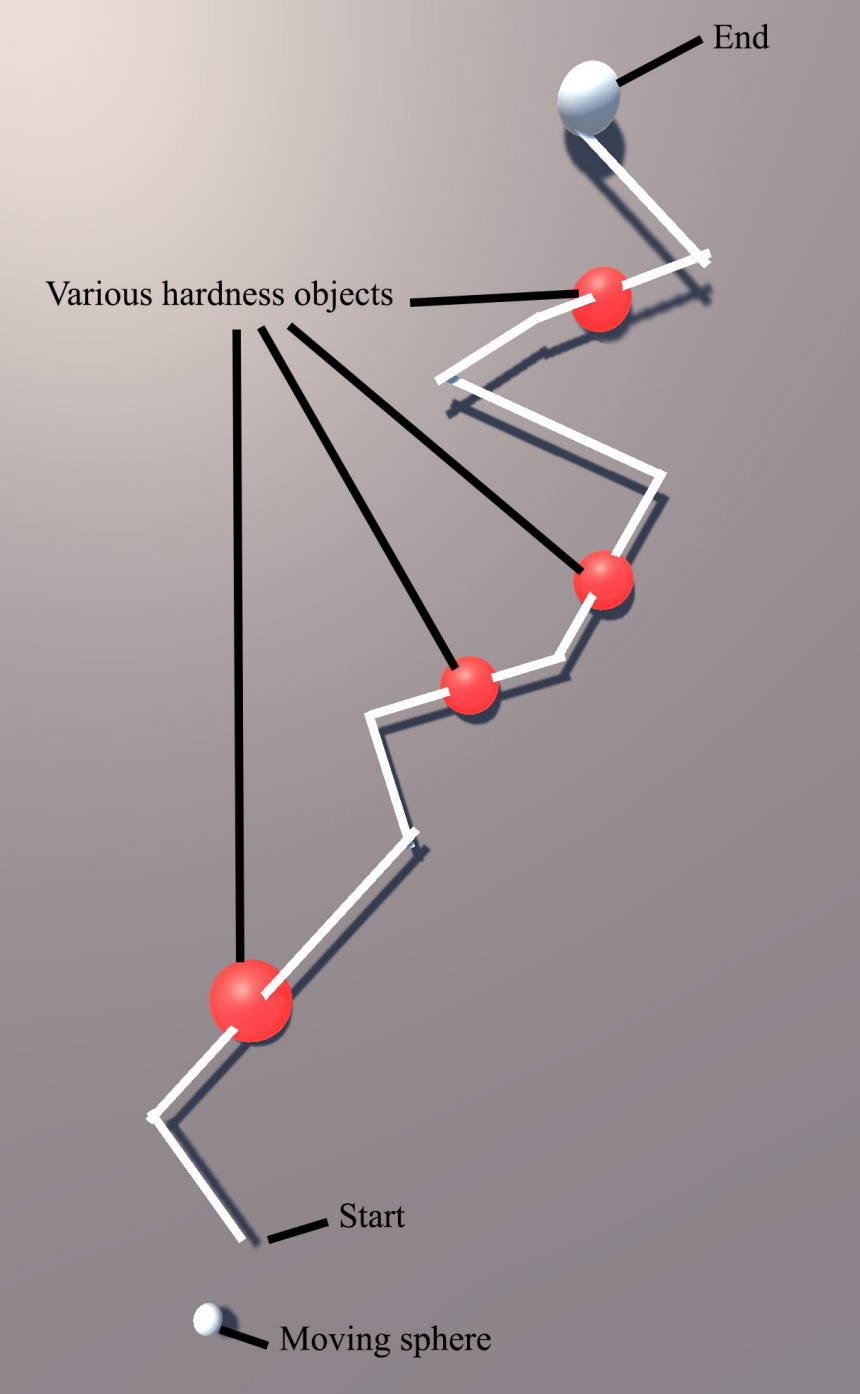
Task in Unity 3D showing a virtual path, moving sphere and lumps of various hardness levels.

Figure 12 shows a snapshot of the environment; it includes a path (white) with start and end with 4 red objects placed at random points on the path (the size of each object has no importance in terms of haptic information). This path was the basis of a user study aimed at the evaluation of the FHD and its effectiveness in determining various levels of hardness as well as effectiveness in distinguishing between a safe and a “no-go zone”. Testing of the two features simultaneously provides a realistic scenario e.g., in a surgical operation where sensory information can be convoluted, and the surgeon must be able to correspond each cutaneous signal to its own stimuli. In total, 14 participants (24–38 years old, ratio of women/men 1:1, ratio of right/left dominant hand 12/2) took part in this study.

Participants were asked to put the FHD on the index finger of their dominant hand, as shown in Figure 1A, and use it to move the small white sphere (bottom part of Figure 12) along the path. They did this by tilting their index finger (pitch) to control the forward/backward movement and pointing in the direction parallel to the sphere’s chosen path (yaw). The IMU tracks the change of direction and the virtual sphere moves accordingly. The goal of the task was to move the sphere from start to end as fast and as accurately as possible, while receiving haptic feedback from the FHD. Force feedback is initiated when the small white sphere derails from the path as well as when it touches a red object (lump). The participants also need to discern which 2 of the 4 red lumps are the hardest.

**Figure 13:**
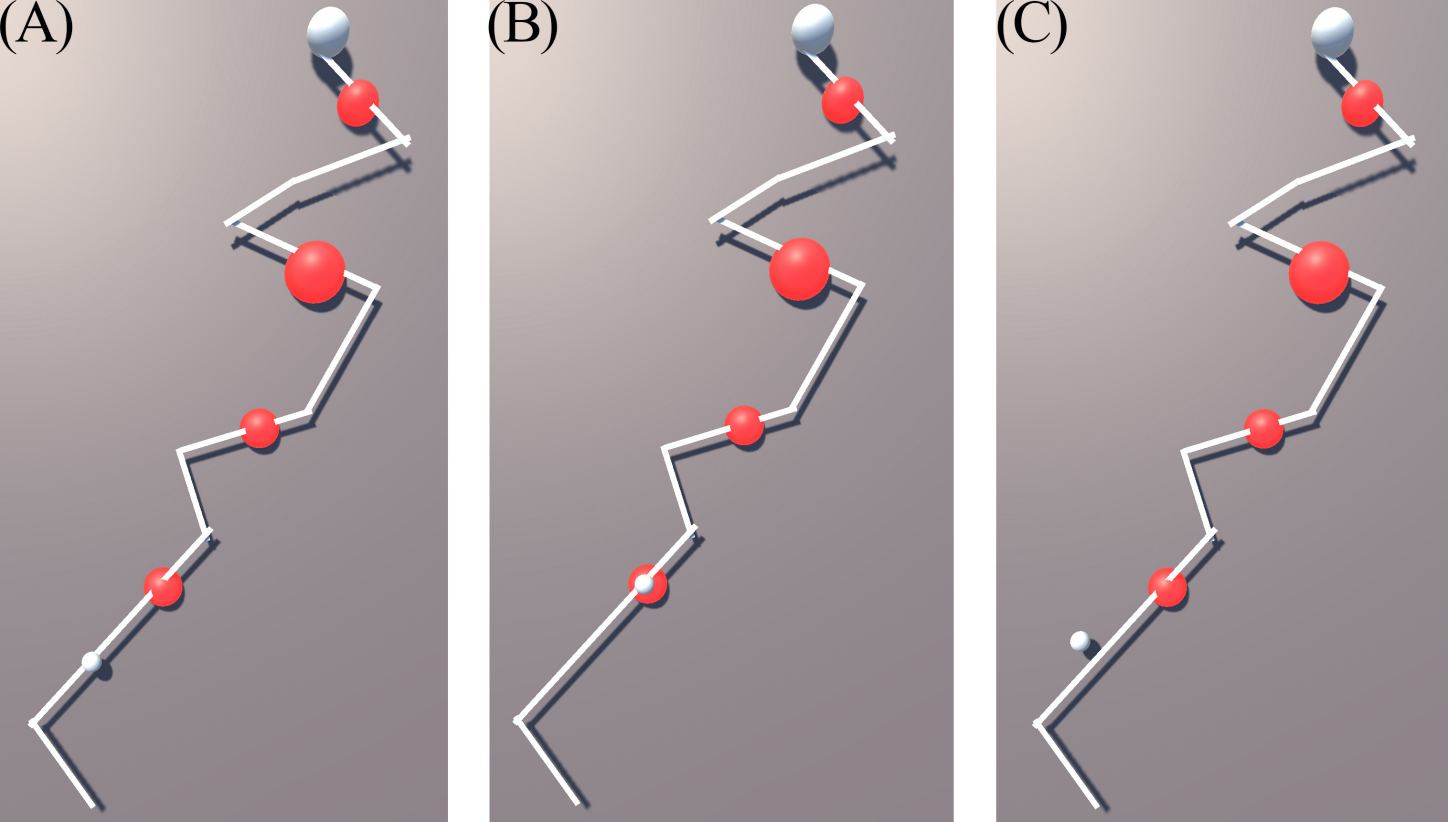
Snapshots from an experiment where the moving sphere is **(A)** on the path, **(B)** on a red lump and **(C)** off-path.

A short “training” session allowed participants to get accustomed with navigation in the VR environment following a path (different to the path of the main experiment) and to familiarize themselves with various hardness levels by interacting with virtual objects. Subsequently, each participant completed 3 sets of a total of 6 tasks in a random sequence. In each of the 6 tasks, the participants experience various levels of haptic feedback when the sphere moves off-path (Figure 13C) and when it touches the red lumps (Figure 13B). The levels of hardness of the red lumps were chosen randomly and are summarised in Table 4. In task 4, the FHD provided no haptic feedback when the sphere derailed from the path or was on the lumps. Furthermore, the area surrounding the path was divided in 3 zones (inner, middle and outer zone), triggering levels of haptic feedback corresponding to increasing hardness as the sphere derails from the path, as shown in Table 4.

**Table 4:** Levels of hardness (1–5 for hard-soft) of (a) the red lumps per experimental path (from the one proximal to the start towards the end of the path) (b) the zones surrounding the path and (c) success rate for detection of the hard red lumps per path.

	Hardness level of red lump				Level of haptic feedback in zones surrounding the path			User success rate in detecting the hard red lumps
	1st	2nd	3rd	4th	Inner	Middle	Outer	
Task 1	“2”	“4”	“3”	“4”	“3”	“2”	“1”	83.3%
Task 2	“3”	“5”	“5”	“3”	“5”	“4”	“3”	80%
Task 3	“3”	“2”	“3”	“2”	“1”	“1”	“1”	78.6%
Task 4	No haptic feedback							n/a
Task 5	“5”	“3”	“3”	“5”	“4”	“3”	“2”	90.5%
Task 6	“3”	“3”	“4”	“4”	“2”	“1”	“1”	84.5%

The duration of each task, how long the moving sphere was off-path and its distance from the path were recorded. The RMS error of the distance between the moving sphere and the path was calculated to evaluate the accuracy and efficiency of the device at each level of haptic feedback. Note that the RMSE was calculated in units of the VR environment which do not correspond to physical units of measurements. For this reason, no units have been used in the analysis of the RMSE metric below.

#### 3.2.1. Detection of Lump Hardness

The success rate of the hard red lump detection for each task is summarised in the last column of Table 4. Participants were most successful in task 5 with a success rate of 90.5%. It is worth noting that tasks 5 and 2 involved red lumps with the same level of hardness (“3” and “5”). However, the levels of haptic feedback for the path differ between the two tasks, with the inner zone represented by “4” and “5” respectively. It is possible that the difference between the red lump hardness and the haptic feedback of the inner zone of task 5 lead to a better perception of the hardness of the lumps.

It can also be seen that successful detection was the lowest during task 3, where the haptic feedback of all zones had a level of “1” (rated as the hardest in the previous study). It is possible that the abrupt change from no feedback (when sphere is on the path) to maximum feedback (sphere goes off-path) confused the participants as they could not distinguish clearly whether the received feedback was related to the lump or the path.

#### 3.2.2. Learning Curve of FHD

To investigate the learning curve related to use of the FHD, the mean success rate of hard red lumps detection was calculated for each set of the 6 tasks (3 sets in total). It was found that there was an overall improvement from 81.4% at the 1st set to 87.1% successful detections at the final set of tasks when participants were more accustomed with the FHD. The same trend is true for other objective metrics, i.e., the RMS error of the distance between the moving sphere and the path, the duration of each trial and the total off-path time. These results are summarised in Table 5, showing 28% increased accuracy in following the path, 23% faster completion of each experiment and 45% reduction in time spent off-path.

**Table 5:** Mean of objective metrics over all participants for each set of the and 6 tasks.

	1st Set	2nd Set	3rd Set
User success rate in detecting the hard red lumps	81.4 %	82.8 %	87.1 %
RMSE in distance from path	1.774	1.636	1.273
Completion Time	41.25 s	33.99 s	31.78 s
Off-path total time	5.5 s	3.74 s	2.26 s
Percentage of off-path total time/completion time	13.23 %	9.66 %	7.33 %

#### 3.2.3. Comparison of tasks by level of haptic feedback

The mean of the same objective metrics can be calculated for each task and these are given in Table 6, where for each metric the best result between the Tasks is highlighted. Task 4 did not include any haptic feedback but instead participants relied solely on vision. Although it was completed in the shortest time, it was the one with the highest RMS error in the distance from the path and the highest percentage of off-path time out of the total completion time. On the contrary, all other tasks were associated with higher accuracy despite a longer completion time. Task 5 had the highest accuracy with a 23% improvement compared to the accuracy without haptic feedback. Furthermore, when haptic feedback was available, time spent off-path was reduced, e.g., by 45% in task 3 and by 36% in task 5.

**Table 6:** Mean of objective metrics over all participants for each task.

	Task 1	Task 2	Task 3	Task 4	Task 5	Task 6
Success rate of detection of hard red lumps	83.3%	80%	78.6%	n/a	90.5%	84.5%
RMSE in distance from path	1.562	1.532	1.41	1.76	1.36	1.74
Completion Time	36.11 s	34.9 s	38.87 s	26.54 s	39.11 s	40.56 s
Off-path total time	3.12 s	3.22 s	3.29 s	3.99 s	4.22 s	3.99 s
Percentage of off-path total time/completion time	8.45%	9.06%	8.01%	14.57%	9.35%	11%

**Figure 14:**
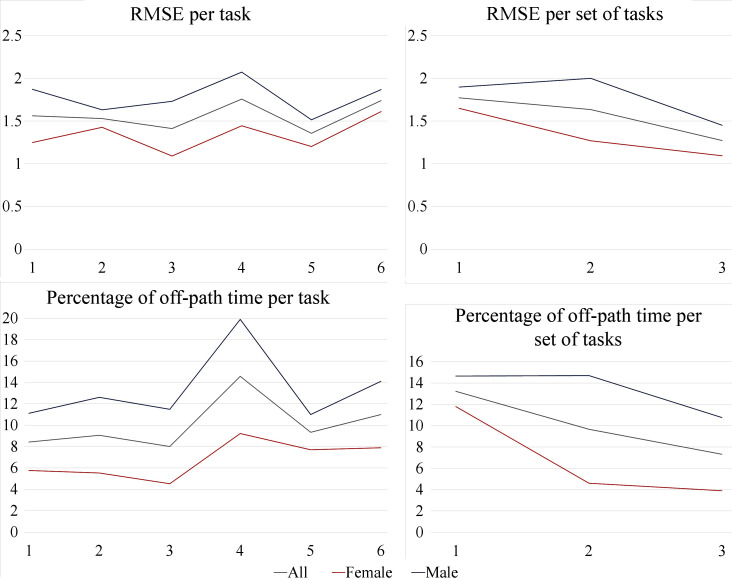
Comparison of RMSE and time off-path between men and women.

A comparison of the results between men and women shows that overall women were more accurate and spent less time off-path (Figure 14). This could be due to differences in fingertip thickness; however, as there were no fingertip measurements taken, this cannot be conclusive.

#### 3.2.4. Experience feedback from the user

After the end of each participation in the study, participants optionally gave feedback on their experience with the FHD and the VR environment, 12 of which answered the following open-style questions:

- Do you have any comments about the “path following” experiment?
- Do you have any comments about the haptic feedback you experienced in general?
- Do you think that haptic feedback helped you complete the task? Do you think you were faster/more efficient?
- You experienced various level of haptic feedback. Do you consider:
  - Any of these levels you experienced as distracting?
  - The levels generally adequate?
  - Any or all levels too small/negligible?

From the 12 respondents answering the feedback form, 1 believed that his performance was better overall without the addition of haptic feedback. However, the objective metrics show that his accuracy in following the path was improved with the presence of haptic feedback. Most respondents thought that the hardness levels were adequate, while 1 thought that continuous haptic feedback was distracting and 1 found the hardest level the most difficult to identify. 7 respondents thought that the haptic feedback they experienced was useful and helped them complete the task, 2 were unsure and 1 found it unhelpful (2 did not comment on this). 4 mentioned that the task was difficult to complete: 2 of these respondents performed close to the average while the other 2 performed above the average in accuracy and without making any mistakes in identifying the lumps’ hardness levels.

When the virtual sphere was on the red lumps, 3 respondents found it difficult to distinguish between haptic feedback due to derailing from the path or due to the lump’s hardness. These respondents made more mistakes in identifying lump hardness level than average, however their path following accuracy was above average. This is also observed from the results of Task 3 in Table 6, where the success of hardness identification is the lowest.

4 respondents mentioned wrist and muscle fatigue during the use of the FHD, but their overall performance was above the average. This was due to the hand movements necessary to control the 3D position of the virtual sphere using data from the IMU. 2 women respondents reported that having thin fingers might have prevented them from appropriately feeling the haptic feedback. Their hardness identification was average and their accuracy following the path was above the average.

## 4. Discussion

The FHD provides haptic feedback via the VCP, a soft deformable surface, which can provide information to the user about objects with variable stiffness, including soft and deformable surfaces. This differs from other works in the literature, where, for example, stiffness is modelled as a rigid spring (Maereg et al., 2017). Furthermore, the FHD can allow for both “pressing” and “tapping” on the user’s fingertip, depending on the task, unlike haptic devices mentioned in the Introduction Section which are continuously in contact with the user’s fingertip.

As noted in Table 2, the FHD can provide a range of forces between 1–7 N. The conditions that were tested had a mean of 4.2 N (SD of 2.1). The range of normal forces in the “robust” conditions had a mean of 3.7 N (SD of 2.3), which is comparable to the 3.2 N mean of normal forces measured when participants palpated tissue using their index finger directly as reported by Konstantinova et al. (2017). The measured forces (of Table 2) are an estimate, as the precise magnitude depends on the thickness of the user’s fingertip. Future work needs to include this measurement as a parameter of the FHD.

The user study for evaluation of the FHD was set up in a way that tests its efficacy on two different components: (a) using the haptic feedback as a warning of going into a “no-go” zone and at the same time (b) identifying lumps of different hardness. In a surgical scenario, the virtual path could represent a part of the human abdomen, while the lumps would represent tissue structures of various hardness. Different hardness can also indicate abnormal tissue, e.g., a tumour. From the results presented in Tables 5, 6, it can be concluded that the presence of haptic feedback improves the positional accuracy of the participants by a mean of 23%, while it also reduces the time spent in the “no-go” zone by 45%. At the same time, participants were able to distinguish between different levels of object hardness, reaching an average of 90.5% success in Task 5.

The conditions that are used in the experiments, and which are described in Table 1, were created by varying the parameters of the FHD and derive the combinations that helped determine its functionality. While these conditions do not relate to specific material properties of objects of daily life or of a surgical scenario, this was necessary so that a characterisation in terms of user perception of softness/hardness was developed. In future work, conditions of the FHD will be mapped to specific objects and this will be further tested by the users.

All participants finished the task faster when no haptic feedback was present. Most participants mentioned this in their feedback form after the experiments but believed that their accuracy was probably worse in that case. The experiments showed that when the VCP was inflated with 3 ml or 4 ml of air and touched the fingertip gently, participants gave the highest scores (indicating feeling of softness). When the displacement of the VCP increased, the hardness of the material was scored lower (harder). Furthermore, an important criterion used in determining the five different levels of hardness was their “robustness”, i.e., conditions that had consistent responses per participant. This was a necessary adaption, since perception of each user for the same condition can be different and due to various physical or psychological factors.

In general, women performed better than men in terms of accuracy, time spent off path and correct identification of “harder” lumps. As mentioned previously, this could be due to the differences in fingertip width or thickness (also reported by respondents on their feedback form), which can be further explored in future work. Calibration in software depending on fingertip in combination with a pressure sensor that detects initial contact with the fingertip width could dissipate these differences. Information from this sensor would be used to adjust the hardness levels; with the current setup, it is possible that users with larger fingertips experience saturation, i.e., levels “1” and “2” feel similar due to the linear displacement of the VCP pushing against their finger too much. Adjusting the linear motion of the VCP according to the user’s fingertip thickness would result in more consistent normal forces among users at each hardness level. Consequently, calibration of the functionality of the FHD according to fingertip size could improve the consistency of user perception for each condition (as summarised in Table 2).

Future work will also include re-designing of the FHD to make it even more compact and portable, as well as replace its tracking system (IMU) with other hand tracking devices such as the sensory hand exoskeleton of our previous work, also meant for application in a surgical scenario (Tzemanaki et al., 2014). Future experiments can include PHF to complement the functionality of the FHD, by showing deformation in the virtual tissues when the user “touches” them as in the work by Li et al. (2015). However, the purpose of this would only serve a scenario e.g. of a simulator for training whereas the FHD could also be able to provide haptic feedback during a surgical procedure.

## Ethics Statement

All experiments were carried out in accordance with the recommendations of the University’s policy on research ethics, UWE Research Ethics Committee. The protocol was approved by the Faculty of Environment & Technology Research Ethics Committee. All subjects gave written informed consent in accordance with the Declaration of Helsinki.

## Author Contributions

AT contributed the majority of the text, made substantial contributions in project conception, design and analysis of data and supervised the development process. GAA made substantial contributions in the design, development of the work and acquisition of data, took part in the analysis of results and contributed to drafting the work. CM contributed to the conception of the work and revised it. SD made substantial contributions to the conception of the work, supervised the design and development and revised the manuscript. All authors approved the final version and agree to be accountable in ensuring that questions related to the accuracy or integrity of any part of the work are investigated and resolved.

## Conflict of Interest Statement

The authors declare that the research was conducted in the absence of any commercial or financial relationships that could be construed as a potential conflict of interest.
